# Serum Concentrations of S100B are not Affected by Cycling to Exhaustion With or Without Vibration

**DOI:** 10.2478/v10078-011-0073-2

**Published:** 2011-12-25

**Authors:** Stefanie Schulte, Thorsten Schiffer, Billy Sperlich, Heinz Kleinöder, Hans Christer Holmberg

**Affiliations:** 1 Institute of Motor Control and Movement Technique, German Sport University Cologne, Cologne, Germany; 2 Institute of Training Science and Sport Informatics, German Sport University Cologne, Germany; 3 Institute of Training Science and Sport Informatics, German Sport University Cologne, Cologne, Germany; 4 Swedish Winter Sports Research Centre, Department of Health Sciences, Mid Sweden University, Östersund, Sweden

**Keywords:** biomarker, neurotrophin, hormone, endurance, serum

## Abstract

The calcium-binding protein S100B is produced primarily by astrocytes and exerts concentration-dependent paracrine and autocrine effects on neurons and glia. The numerous findings of a correlation between S100B and traumatic brain injury (TBI) have resulted in the employment of this protein as a clinical biomarker for such injury. Our present aim was to determine whether cycling with (V) or without (NV) vibration alters serum concentrations of S100B. Twelve healthy, male non-smokers (age: 25.3±1.6 yrs, body mass: 74.2±5.9 kg, body height: 181.0±3.7 cm, VO_2peak_: 56.9±5.1 ml·min^−1^·kg^−1^ (means ± SD)) completed in random order two separate trials to exhaustion on a vibrating bicycle (amplitude 4 mm and frequency 20 Hz) connected to an ergometer. The initial workload of 100 W was elevated by 50 W every 5 min and the mean maximal period of exercise was 25:27±1:30 min. The S100B in venous blood taken at rest, immediately after the test, and 30, 60 and 240 min post-exercise exhibited no significant differences (p>0.05), suggesting that cycling with and without vibration does not influence this parameter.

## Introduction

Serum concentrations of the S100B protein provide a well-established indicator of alterations in the function of the central nervous system (CNS). This protein is expressed primarily by astrocytes and Schwann cells, but also at lower concentrations by other human tissues, such as bone, muscle, heart and adipose tissue (Zimmer and van Eldik, 1987). S100B is involved in promoting axonal growth, the protection and proliferation of glia cells and neuronal differentiation (Kleindienst et al., 2007). The healthy brain does not release S100B into the peripheral circulation to any considerable extent (Marchi et al., 2004; Reiber, 2003). Physiological serum S100B is assessed to be smaller than 0.1 μg·L^−1^ (Cheuvront et al. 2008). However, several investigations have detected elevations in serum S100B following participation in various sports that involve mechanical impact, such as boxing, submaximal endurance like marathon running or swimming, and short but high intensity activities like sprinting (Otto et al., 2000; Hasselblatt et al., 2004; Dietrich et al., 2003). There are contradictory opinions concerning the intensity of physical activity which can lead to a significant increase of S100B in serum. Hasselblatt et al. (2004) suggest a muscular origin due to myofibrillar injury after marathon running. Some researchers have suggested that the influence of exercise on the CNS could be associated with either changes in serotonin levels and its receptors which increases intracellular content and release of astrocytic S100B (Dietrich et al., 2003) and/or increased BBB permeability (Watson et al., 2006).

Vibration training improves strength and flexibility, while at the same time exerting influences on endocrine, metabolic, cardiovascular and central nervous system (Griffin, 1996; Bosco et al., 2000; Korving et al., 2006; Mester et al., 2006). Several reports have pointed out possible damage to human peripheral nerves, blood vessels or perceptual functions as a result of exposure to vibrations (Novotny and Usher, 1959; Manisfield, 2005). Traumatically induced brain tissue damage is associated with increased serum S100B which reflects the presence and severity of brain tissue damage (Stalnacke et al., 2008) and which is established as a biomarker in clinical settings.

Recently, acute cardio-respiratory, metabolic and angiogenic responses to endurance exercise in combination with vibration have been examined (Suhr et al., 2007; Sperlich et al., 2009). For this purpose the bottom crank of a bicycle, was fitted onto a vibration platform and the frame disconnected from any vibrating stimulus in order to direct the shock waves solely onto the lower body muscles. Although such cycling with vibration provides intramuscular benefits, possible associated damage to the brain or peripheral tissue after cycling might be reflected in elevated serum S100B. The aim of our study was to examine if (i) cycling with graded intensities and vibration does result in an elevation of serum S100B and if so (ii) does the increase leave the range of physiological reference values in healthy adults?

## Material and methods

### Participants

Prior to their participation, 12 healthy, male non-smoker (age: 25.3±1.6 years, body mass: 74.2±5.9 kg, body height: 181.0±3.7 cm, VO_2peak_: 56.9±5.1 ml·min^−1^·kg^−1^ (mean±SD) received written information about this study which was approved by the ethics committee of the German Sport University, Cologne, and gave their written informed consent. None of these subjects had any noteworthy medical history, especially with respect to neurological problems. The participants were familiarized in advance with the laboratory procedures and were asked on the test days to arrive well hydrated and at least 2 h after a light meal and not to have performed any strenuous exercise during the 24 h period immediately prior to testing.

### Experimental Procedures

As described in detail elsewhere (Sperlich et al., 2009), the subjects performed an incremental test on a vibration bicycle (Enformax) at 70 rpm, and an initial workload of 100 Watt (W) that was increased by 50 W every 5 minutes until exhaustion. Criteria for exhaustion were a) plateau in oxygen uptake (an increase less than 1.0 ml·min-1 kg-1 despite an increase in power output, b) respiratory exchange ratio greater than 1.10, (c) heart rate ±5% of age predicted maximal heart rate, (d) maximal capillary blood lactate concentration after exercise greater than 6 mmol·L-1 (e) RPE rating greater than 18. In all cases, at least three of the criteria were met. Each participant performed two trials, one with (V) and one without (NV) vibration in random order. The bicycle was connected to a Cyclus 2 Ergometer (Avantronic, Leipzig, Germany) and vibration (at a frequency of 20Hz and amplitude of 4mm) was applied vertically through the crank onto the pedals and from there onto the foot. To insure adequate recovery, the trials were separated by at least 72h but not longer than 96 h. The participants were instructed not to exercise during this period. Blood samples were collected from the ear lobe in a capillary tube (Eppendorf, Hamburg, Germany) and analysed amperometricenzymatically for capillary blood lactate (LA) at the end of each trial utilizing an Ebio Plus Analyser (Eppendorf, Hamburg, Germany). Venous blood samples were drawn at rest, immediately prior to and after the test as well as 30, 60 and 240 min post-exercise. Serum prepared from these samples by centrifugation was frozen and stored at −40° C until analysis for S100B with a luminometric assay (Sangtec®100, DiaSorin, Dietzenbach, Germany).

### Statistical Analysis

All data are presented as conventional mean values and standard deviations (SD) and a check for normality confirmed that no further transformation was required. Repeated-measures ANOVA with the factors CONDITION (V and NV) and TIME (pre, post, +30, +60, +240) was applied to analyse S100B values, while HR and LA were analysed pre vs. post only. An alpha of p<0.05 was used for statistical significance.

## Results

**Figure 1:**
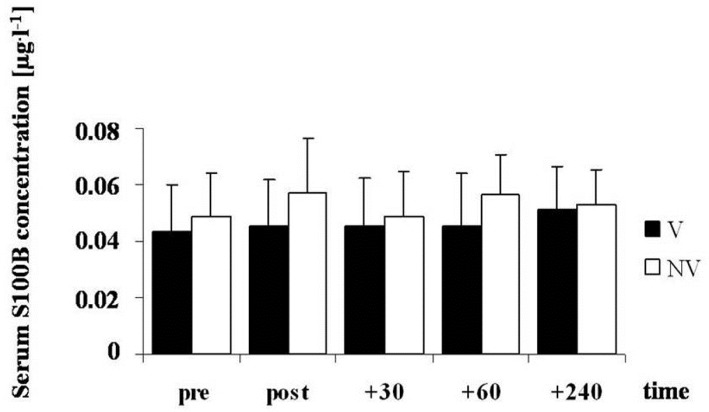
Serum concentrations of S100B pre- and immediately, 30, 60 and 240 min post-exercise with (▪) and without (□) vibration. In all cases, p<0.05

The average maximal exercise time was 25:27±1:30 min and there were no statistically significant differences for CONDITION in heart rate (pre 78 (5.4) versus 76 (3.2); post 177.2 (7.2) versus 182.4 (8.9) bpm) [F (1, 10) = 0.001 p = 0.753] or LA (pre 0.7 (0.3) versus 0.8 (0.3) mmol·L^−1^; post 7.8 (2.8) versus 8.2 (2.4) mmol·L^−1^) [F (1, 10) = 0.200, p = 0.645] in the V and NV trials, respectively. For TIME ANOVA revealed significant differences between pre and post values for heart rate [F (1, 10) = 1279, p < 0.001] and LA [F (1, 10) = 121.6, p < 0.001]. The baseline serum S100B (mean = 0.05, range 0.01– 0.09 μg·L^−1^) was within the normal range for adult subjects. The ANOVA yielded no significant effects for CONDITION [F (1, 10) = 1.499, p = 0.249], for TIME [F(4, 40) = 1.425, p = 0.243] or for their INTERACTION [F (4, 40) = 0.947, p = 0.446] (Figure 1).

## Discussion

The present findings indicate that serum concentrations of S100B are not affected by incremental cycling to exhaustion with or without vibration. To our knowledge, no such investigation has been reported previously. However, alterations in serum S100B have been observed following participation in various sports involving rigorous mechanical impact on the head, such as boxing, and/or sudden changes in direction, jumps and/or physical impact, as in basketball, soccer and hockey (Otto et al., 2000; Stalnacke et al., 2003; 2004; 2006). Such findings suggest that exercise may cause mechanical damage to the brain cells with a subsequent release of S100B.

Since the responsible mechanisms for passage of S100B through the blood-brain barrier are mainly believed to be through passive diffusion, we can only speculate that the lack of alterations in serum S100B observed here were due to a missing influence on the brain cells. First of all, the vibration was applied onto the leg muscles, not directly to the head. In the case of contact sports such as soccer, rough physical contact to the head is associated with elevations in serum S100B (Stalnacke et al., 2006). Apparently, no harmful impact to the head was associated with the present test design.

Furthermore, physical activity to exhaustion exerts both acute and chronic influences on neurotransmission (Awga et al., 2008; Strueder and Weicker, 2001). As a neurotrophic factor, S100B regulates various signal transduction cascades that result in stimulation of, for example, serotonergic neurons (Nishi et al., 2000, Nishiyama et al., 2002) and, moreover, expression of this protein is regulated in part by serotonin transmission and serotonin receptors (Rothermundt et al., 2003). Since, alterations in serum S100B are only apparent following physical activity to exhaustion or after competition for approximately 2 hours (Strueder and Weicker, 2001; Dietrich et al., 2003), the approximately 30-minute period of the exercise employed here might have not been long enough to cause such alterations.

## Conclusion

The current investigation indicates that cycling with and without vibration according to our test protocol is not associated with alterations in serum [S100B] and can be employed to obtain the known intramuscular benefits without damage to the brain, even when vibration is applied.
